# Supplementary material for: Connecting shear localization with the long-range correlated polarized stress fields in granular materials
Source: Nat Commun. 2020 Aug 28;11:4349. doi: 10.1038/s41467-020-18217-x (PMC7455740; doi:10.1038/s41467-020-18217-x)
Supplement: Supplementary file 1 — Supplementary Information [file 41467_2020_18217_MOESM1_ESM.pdf]

Supplementary Information: Connecting shear localization with the long-range correlated polarized stress fields in granular materials

Wang et al.

Supplementary Figures

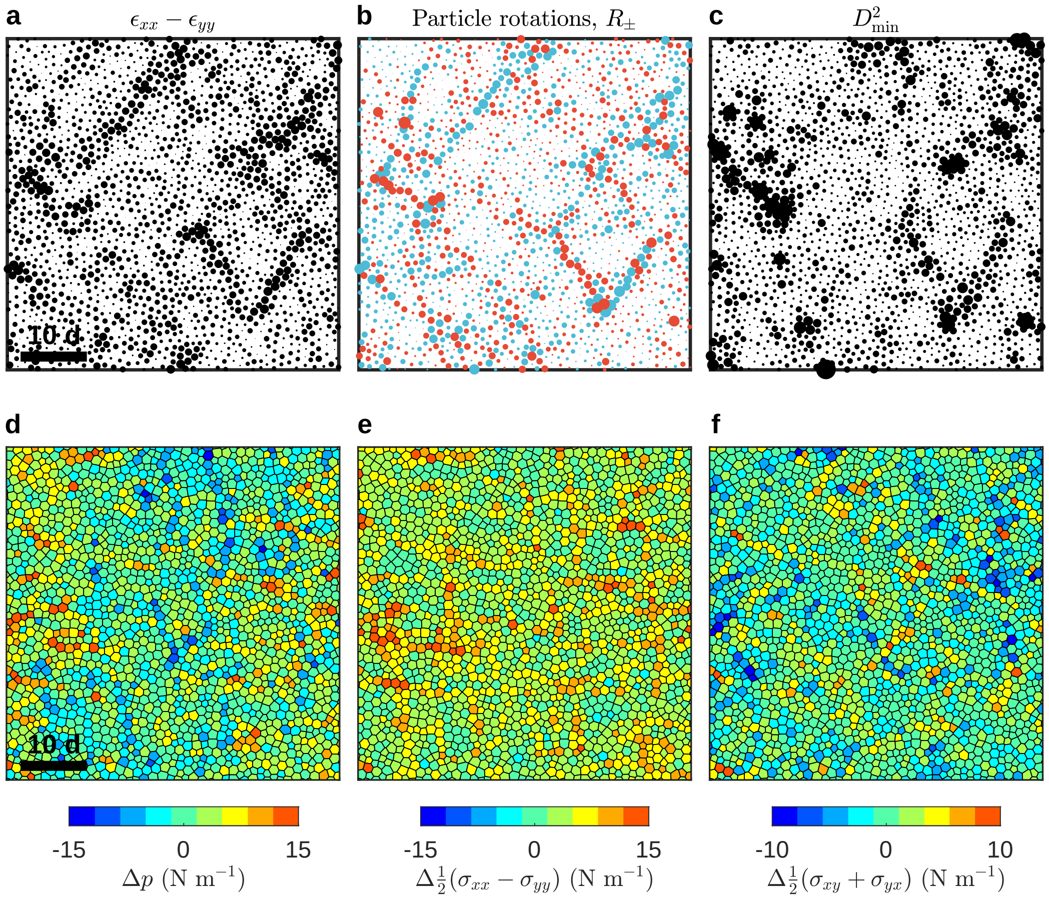

Supplementary Figure 1. Shear localization and particle-scale stress changes. Spatial distributions of **a**, local shear strain $\epsilon_{xx} - \epsilon_{yy}$, **b**, particles rotations (blue circles indicate clockwise rotations, red circles indicate counterclockwise rotations) and **c**, $D^2_{\min}$. Spatial distributions of particle-scale stress changes for three components **d**, $p = \frac{1}{2}(\sigma_{xx} + \sigma_{yy})$, **e**, $\frac{1}{2}(\sigma_{xx} - \sigma_{yy})$, **f**, $\frac{1}{2}(\sigma_{xy} + \sigma_{yx})$. All of these quantities are measured from $\gamma = 0\%$ to $\gamma = 0.75\%$, the same as those in main text. Scale bar = 10 d, where d is the diameter of small particle.

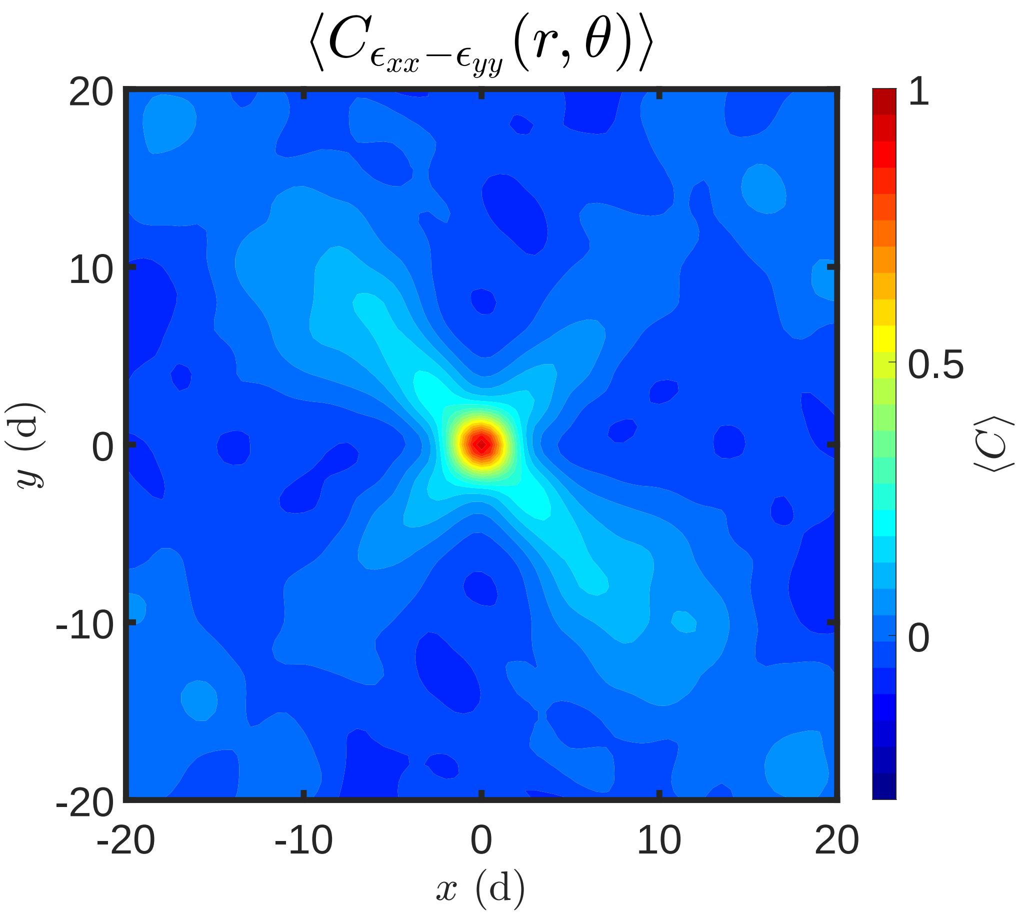

Supplementary Figure 2. Autocorrelation map of local shear strain $\epsilon_{xx} - \epsilon_{yy}$. The local shear strain is measured from $\gamma = 0\%$ to $\gamma = 0.75\%$. The map is averaged over six independent runs. $\langle C\rangle$ denotes the correlation function.

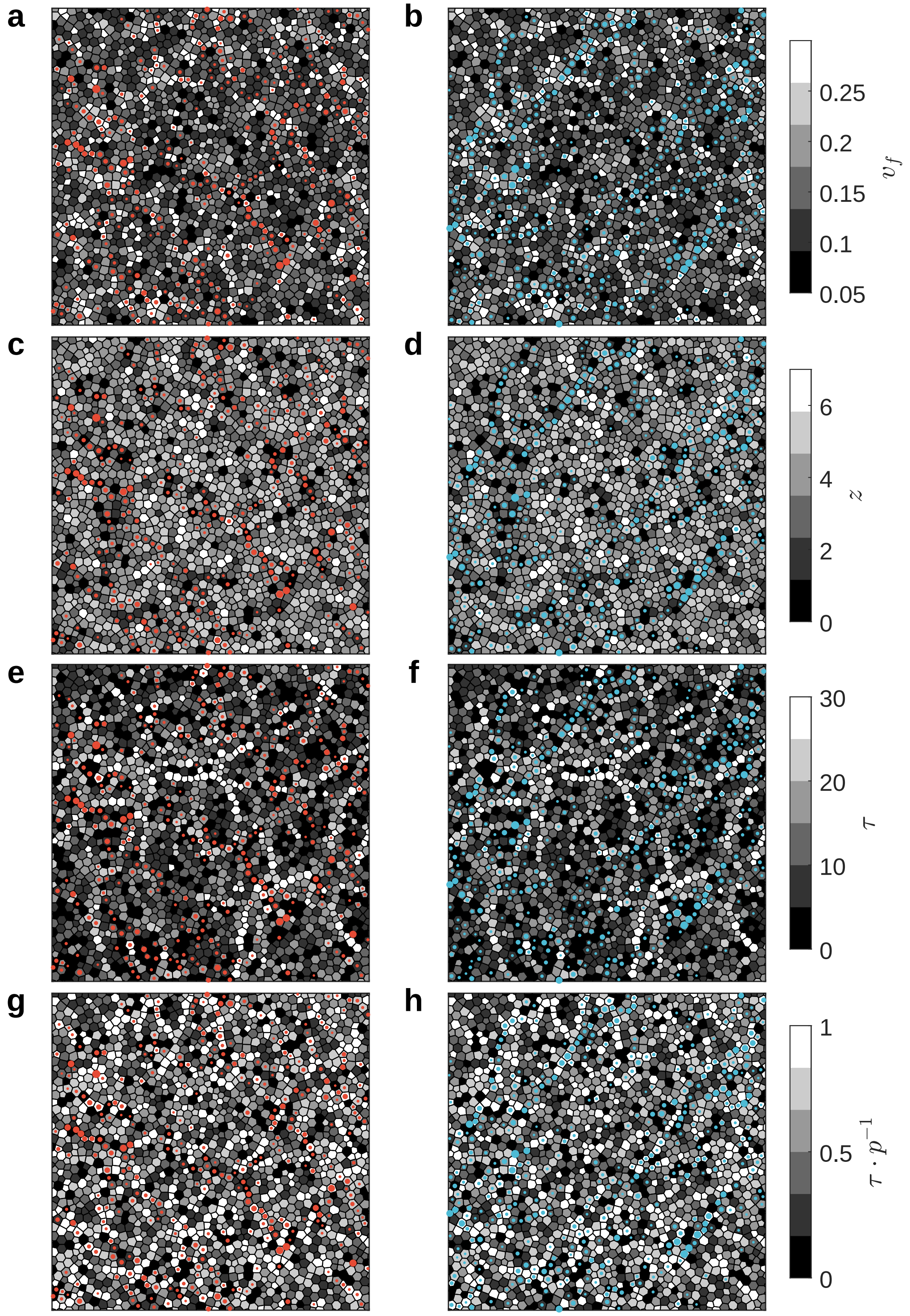

Supplementary Figure 3. Correlations between particle rotations and other quantities. **a**, For counterclockwise rotations R_+ and free volume $v_f = (S_i - \pi r_i^2) \cdot (\pi r_i^2)^{-1}$, S_i is the voronoi area of disk i and r_i is its radius. $C(v_f, R_+) = -0.34$. **b**, For clockwise rotations R_- and v_f. $C(v_f, R_-) = -0.19$. **c**, For R_+ and contact number z. $C(z, R_+) = 0.15$. **d**, For R_- and z. $C(z, R_-) = 0.15$. **e**, For R_+ and deviatoric shear stress τ (one half of the difference between two eigenvalues of a stress tensor). $C(\tau, R_+) = 0.08$. **f**, For R_- and τ. $C(\tau, R_-) = 0.16$. **g**, For R_+ and the ratio of the deviatoric shear stress over pressure $\tau \cdot p^{-1}$. $C(\tau \cdot p^{-1}, R_+) = -0.15$. **h**, For R_- and $\tau \cdot p^{-1}$. $C(\tau \cdot p^{-1}, R_-) = -0.13$.

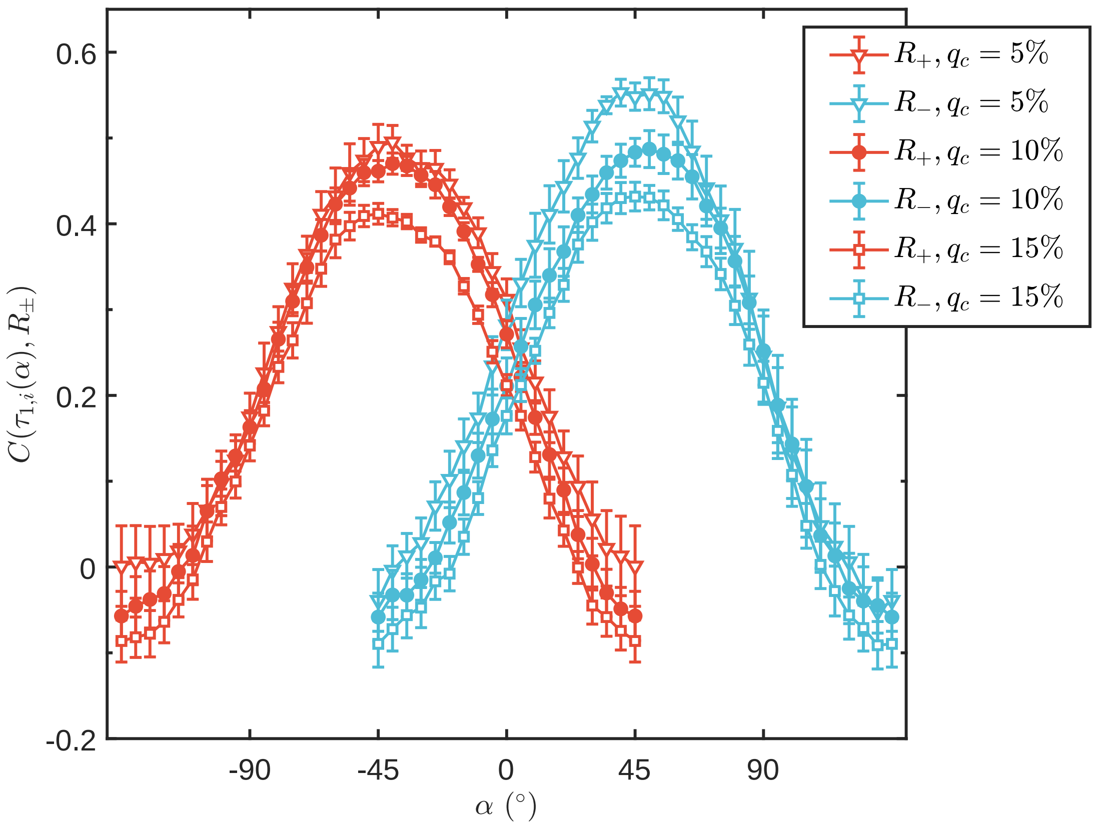

Supplementary Figure 4. Correlations between particle rotations and $\tau_{1,i}(\alpha)$ versus angle α, for different cutoffs q_c of particle rotations.

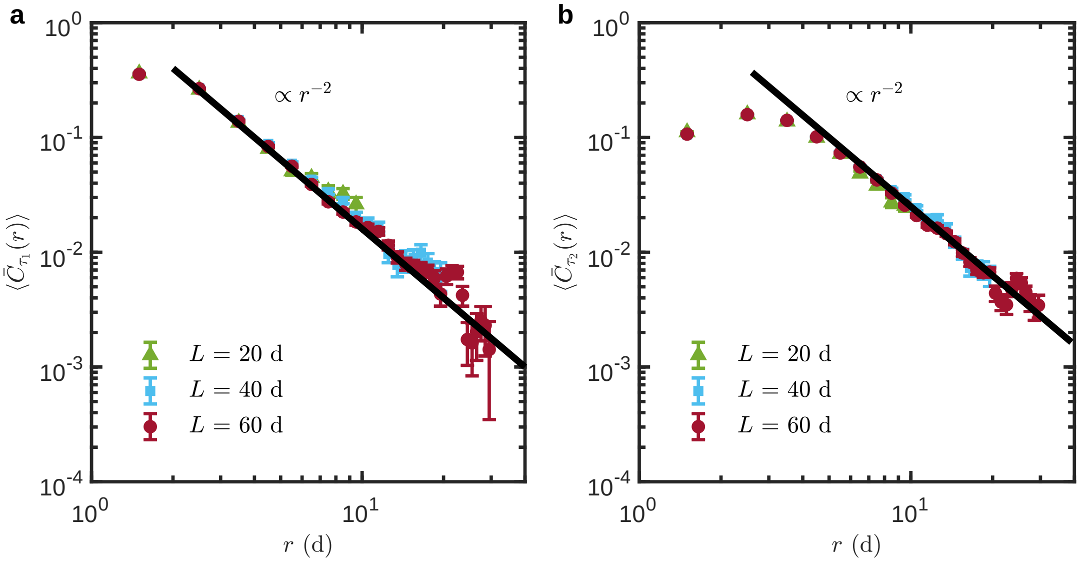

Supplementary Figure 5. Finite size analyses of stress correlation functions. Finite size analyses of angle-averaged correlation functions **a**, $\langle \bar{C}_{\tau_1}(r) \rangle = \pi^{-1} \int_0^{2\pi} \mathrm{d}\theta \cos(2\theta) \langle C_{\tau_1}(r, \theta) \rangle$ and **b**, $\langle \bar{C}_{\tau_2}(r) \rangle = \pi^{-1} \int_0^{2\pi} \mathrm{d}\theta \cos(4\theta) \langle C_{\tau_2}(r, \theta) \rangle$, for system sizes of L = 20 d, 40 d and 60 d. The black lines indicate a power law of r^{-2}, as a guide to the eye.
